# The Efficacy and Safety of Intravenous Colistin Plus Aerosolized Colistin Versus Intravenous Colistin Alone in Critically Ill Trauma Patients With Multi-Drug Resistant Gram-Negative Bacilli Infection

**DOI:** 10.7759/cureus.49314

**Published:** 2023-11-23

**Authors:** Loveleen Maan, Neelesh Anand, Ghanshyam Yadav, Manjaree Mishra, Munesh K Gupta

**Affiliations:** 1 Anaesthesiology and Critical Care, Institute of Medical Sciences, Banaras Hindu University, Varanasi, IND; 2 Anaesthesiology, Institute of Medical Sciences, Banaras Hindu University, Varansi, IND; 3 Anaesthesiology, Institute of Medical Sciences, Banaras Hindu University, Varanasi, IND; 4 Microbiology, Banaras Hindu University, Varanasi, IND

**Keywords:** critically - ill patients, colistin, ventilator associate pneumonia (vap), gram negative bacteria (gnb), multi-drug resistant (mdr)

## Abstract

Background and aim: Gram-negative bacteria (GNB) with potential multiple drug resistance (MDR) have emerged as a major group of organisms causing ventilator-associated pneumonia (VAP). Higher concentrations are deposited directly in the lungs when antibiotics are given via inhalation, minimizing systemic side effects. This study aims to compare the efficacy and safety of intravenous plus aerosolized colistin versus intravenous (IV) colistin alone in critically ill trauma patients who reported MDR-GNB infection on endotracheal aspirate culture.

Methods: A hundred patients were recruited in the Intensive Care Unit, Trauma Centre, Institute of Medical Sciences, Banaras Hindu University, Varanasi, and randomly assigned to the control (n=50) group, which received IV colistin plus aerosolized colistin and the intervention group (n = 50), which received IV colistin alone. Changes in total leucocyte count (TLC), renal function test (RFT), endotracheal aspirate culture, 24-hour urine output, length of ICU stay, and 28-day ICU mortality were investigated.

Results: Patients receiving intravenous plus nebulized colistin therapy had a better outcome compared to IV colistin alone in terms of faster eradication of MDR-GNB infection. A rise in serum urea and creatinine levels was seen in both groups, which were significantly higher, along with a decrease in urine output in the group receiving intravenous colistin alone. No significant difference was observed in serum sodium and potassium levels in the RFT protocol, length of ICU stay, or 28-day ICU mortality.

Conclusion: Intravenous nebulized colistin could be considered a better alternative therapy for VAP caused by multi-drug-resistant Gram-negative bacteria in the ICU in terms of faster microbiological cure and lesser nephrotoxicity.

## Introduction

The prevalence of nosocomial infections has been on the rise due to increasing multi-drug resistance in Gram-negative bacteria (GNB), which poses challenges in intensive care units. Ventilator-associated pneumonia (VAP) is defined as pneumonia occurring in critically ill patients 48 hours after the initiation of mechanical ventilation [1]. Depending on the person's immunocompetency, VAP may result from a wide range of bacterial pathogens or, rarely, viral or fungal origins.

GNB with potential multiple drug resistance (MDR), e.g., *Acinetobacter* spp., *Pseudomonas aeruginosa*, Enterobacter spp., and *Klebsiella pneumoniae*, are among the most common organisms associated with VAP. Some studies have also reported an increase in methicillin-resistant *Staphylococcus aureus* as the main organism causing VAP in intensive care units [1].

The paucity of novel antibiotics has forced clinicians to reconsider some "old" antibiotics, such as colistin (polymyxin E). Koyama discovered colistin, or polymyxin E, which has been used to treat infections caused by Gram-negative bacteria. However, side effects of colistin, such as nephrotoxicity and neurotoxicity, were significant and its use declined. Safer and other effective antibiotics, such as cephalosporins, amikacin, etc., were preferred.

Due to inadequate lung penetration of colistin, the optimal colistin dose for the treatment of VAP is not very clear [2]. To overcome this limitation, the American Thoracic Society and Infectious Diseases Society of America suggested adjunctive aerosolized colistin for patients with highly resistant organisms or for patients who are not responding to intravenous (IV) antibiotics alone [3]. To attain therapeutic concentration in smaller airways, the dose of colistin required would produce systemic toxicity. Higher concentrations are deposited directly in the lungs when antibiotics are given via inhalation, as well as minimizing systemic side effects [4]. Inhalation therapy has the capability of directly targeting the airways, creating increased and more sustained local concentrations, thereby increasing the therapeutic index, improving efficacy, minimizing toxicities, and decreasing the time of onset for the administered drug. The large alveolar surface area and the thin epithelial layer of the lungs allow an extra edge for inhaled compounds over IV administration, as lung penetration after the latter is erratic and insufficient [5]. Hence, inhalation of colistin can prove to be a better alternative, as it lowers systemic side effects and maintains high concentrations of the drug in the lungs [6]. Previous research has demonstrated that following the inhalational therapy of colistin, susceptible *Acinetobacter baumannii* strains were eliminated [7]. Inhaled colistin is now being efficiently used for treating Pseudomonas aeruginosa infections in cystic fibrosis patients [8].

In this study, we evaluated and compared the efficacy and safety of intravenous plus aerosolized colistin and intravenous colistin alone in critically ill trauma patients who reported MDR-GNB infection on endotracheal aspirate culture.

## Materials and methods

This study was conducted at the Intensive Care Unit, Trauma Centre, Institute of Medical Sciences, Banaras Hindu University, Varanasi. After approval from the ethical committee and obtaining written informed consent from the patient’s attendants, 100 patients were enrolled in a double-blinded prospective randomised control trial (RCT). Both the participant and the investigator were blinded. Randomization was done using computer-generated software. The trial was registered with the Clinical Trials Registry - India (ICMR-NIMS): CTRI/2019/03/017941.

Hundred critically ill poly-trauma patients of both sexes between the ages of 18 and 65, requiring ICU and mechanical ventilation, who reported positive organisms for MDR Gram-negative bacteria in endotracheal aspirate culture were included in this study for 14 days. The culture was determined in patients with a duration of mechanical ventilation >48 hours and clinical and radiological signs of VAP. MDR was defined as an isolate that was resistant to at least one antibiotic in three or more drug classes. Patients with chest injuries, morbid obesity, uncontrolled comorbidities, hemodynamic instability, spine injuries, fungi, and Gram-positive bacteria in the endotracheal aspirate were excluded from the study. These 100 patients were equally and randomly allocated into two groups of 50 patients each, using computer software.

Blood samples were drawn from a peripheral vein, preferably an antecubital vein, for the assessment of the total leucocyte count (TLC) and renal function test (RFT), respectively.

The endotracheal aspirate was collected in a sterile container using a sterile suction catheter and sent for baseline culture. Twenty-four hours of urine output were monitored. A nasogastric tube was used to provide enteral feeding, as advised by the dietitian. The right subclavian vein was the preferred site for central venous catheter insertion, and appropriate prophylaxis for the prevention of deep vein thrombosis was given. Head-of-bed end elevation of 30-45º, comprehensive oral care, and routine endotracheal suctioning were part of the protocol for the prevention of VAP. Complete physical hygiene was maintained. Routine chest and limb physiotherapy was performed for the prevention of decubitus ulcers. Percutaneous tracheostomy was performed in patients requiring long-term mechanical ventilation.

Patients in group 1 were given colistin as a loading dose of 9 MIU diluted in 100 mL of 0.9% saline over 30 minutes intravenously, followed by 3 MIU diluted in 100 mL of 0.9% saline over 30 minutes, every 12 hours at 6 a.m. and 6 p.m. for 14 days. Nebulization with 2 MIU of colistin diluted with 5 mL of sterile 0.9% saline was done immediately via a compressor nebulizer for 30 minutes or until the nebulizer chamber was empty every 12 hours.

Patients in group 2 were given colistin as a loading dose of 9 MIU diluted in 100 mL of 0.9% saline over 30 minutes intravenously, followed by 4.5 MIU diluted in 100 mL of 0.9 saline over 30 minutes, every 12 hours at 6 a.m. and 6 p.m. for 14 days. Nebulization with 5 mL of sterile 0.9% saline was done via a compressor nebulizer for 30 minutes or until the nebulizer chamber was empty every 12 hours.

On the inspiratory limb proximal to the Y piece, the nebulizer was positioned. Ventilator settings were adjusted so as to reduce turbulence in flow and extra-pulmonary deposition. They included the removal of conventional humidifiers, volume-controlled mode, administration of constant inspiratory flow, a respiratory rate of 12 breaths/min, an inspiratory expiratory ratio of 50%, a tidal volume of 8 ml/kg, and an end-inspiratory pause representing 20% of the duty cycle. During the nebulization period, expired aerosol particles were collected in a filter with a pore size equal to 0.2 µm positioned on the distal part of the expiratory limb.

Proper instructions with a checklist were given to the nurses regarding the nebulization of colistin and giving IV colistin, and a protocol was attached to the patient’s bedside for awareness and the nurse’s compliance with the protocol.

After the baseline endotracheal aspirate culture, which reported multi-drug-resistant Gram-negative bacteria, further cultures were sent on the 5th, 10th, and 14th days. A total leucocyte count and renal function test were performed on days 0, 5, 10, and 14 after starting the therapy. Monitoring of urine output was done daily for 14 days, and any decline was noted. Results were compared between groups 1 and 2 based on microbiological eradication as the primary outcome and nephrotoxicity, length of ICU stay, and 28-day ICU mortality as secondary outcomes.

The sample size was calculated based on the presumption that the minimum expected difference between the two groups was 25%. For statistically significant results with α=0.05 and β=0.80, 50 patients were required in each group [Fleiss, Statistical Methods for Rates and Proportions]. Results are presented as mean ± standard deviation (SD) for continuous variables and frequency with their respective percentages for categorical variables. For categorical data, the chi-square test and the Fischer exact test were used. For paired samples, a paired Student's test was used. P-values less than 0.05 were considered statistically significant. The data were extracted and analysed using SPSS version 27 (IBM Corp., Armonk, NY).

## Results

**Figure 1:**
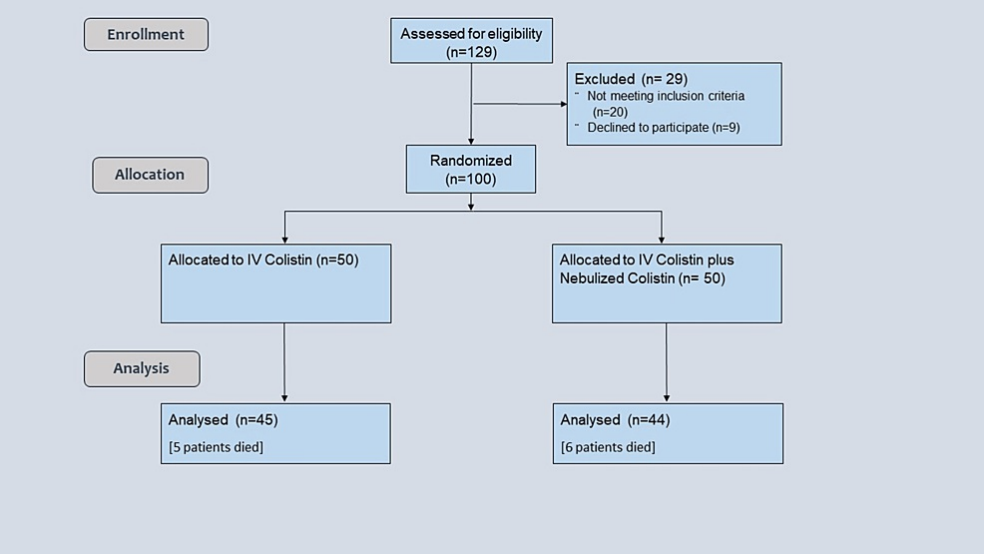
Flowchart of participants through the study n = number of patients

**Figure 2:**
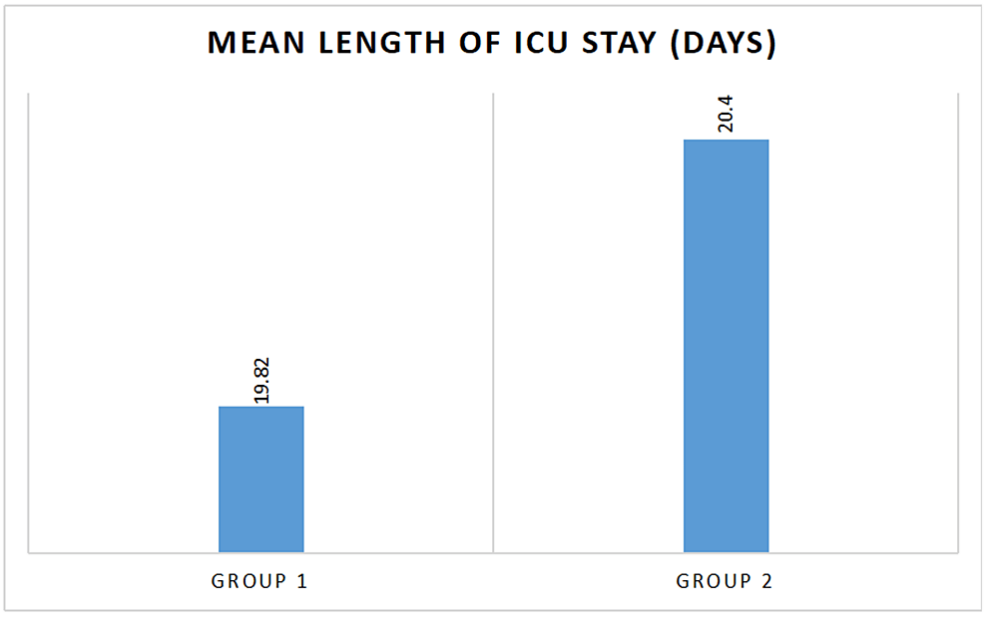
Comparison of mean length of ICU stay

**Figure 3:**
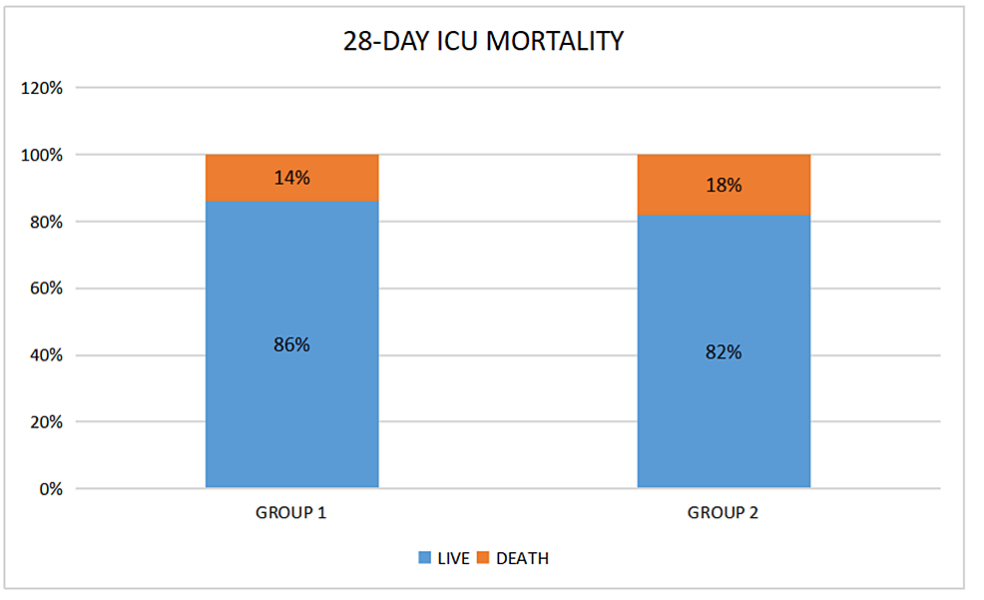
Comparison of 28-day ICU mortality

Among a total of 129 patients, 29 were excluded and 100 were finally included in the study, out of which 16 died during the study (Figure 1). There was no significant difference between the two groups regarding patient characteristics (p = 0.64; 0.33; 0.29; 0.52) (Table 1) and baseline parameters, which included APACHE II score (p = 0.28), RFT (p = 0.18), TLC (p = 0.22), endotracheal aspirate culture (p = 0.99; 0.80; 0.99), and 24-hour urine output (p = 0.89) (Table 2). The control group showed a significant decline in urine output (p = 0.72; 0.06; 0.01) along with an increase in serum urea (p = 0.17; 0,04; 0.02) and serum creatinine (p = 0.25; 0.03; 0.02) levels, as well as a significantly lowered TLC (p = 0.001; <0.001; 0.33) and a significant reduction in the incidence of purulent secretions and positive endotracheal culture reports (p = 0.03; <0.001; 0.33). This was also noted in the intervention group on day 5 (Table 3), day 10 (Table 4), and day 14 (Table 5). However, we could not observe any significant difference among the groups regarding serum sodium (p = 0.28; 0.06; 0.06) and potassium levels (p = 0.27; 0.34; 0.46) and length of ICU stay (p = 0.65) (Figure 2) and 28-day mortality (p = 0.83) (Figure 3).

**Table 1:** Patient characteristics

Parameters		GROUP 1 (n=50) (mean ± SD)	GROUP 2 (n=50) (mean ± SD)	P-value
Age (years)		56.14 ± 10.63	55.06 ± 12.20	0.64
Weight (kg)		68.57 ± 12.65	69.19 ± 9.80	0.33
Height (cm)		152.23 ± 6.50	156.37 ± 7.18	0.29
APACHE II Score		16.26 + 2.149	16.29 + 1.836	0.28
Gender	Male	34 (68%)	37 (74%)	0.52
	Female	16 (32%)	13 (26%)	

**Table 2:** Baseline assessment of parameters at day 0 n: number of patients, SD: standard deviation, TLC: total leucocyte counts

Parameters		Group 1 (n=50) (mean ± SD)	Group 2 (n=50) (mean ± SD)	P-value
TLC (× 10^3^/mm^3^)		21.91 ± 1.88	22.49 ± 2.759	0.22
Serum urea (mg/dL)		40.92 ± 8.88	43.62 ± 10.87	0.18
Serum creatinine (mg/dL)		0.75 ± 0.12	0.83 ± 0.23	0.18
Serum Na^+ ^(mmol/L)		140.22 ± 4.94	139.46 ± 4.40	0.42
Serum K^+ ^(mmol/L)		4.13 ± 0.30	4.06 ± 0.41	0.37
24-hour urine output (mL)		1829.60 ± 252.55	1837.40 ± 308.23	0.89
Endotracheal culture	Acinetobacter baumannii	32 (64%)	33 (66%)	0.99
	Klebsiella pneumoniae	11 (22%)	9 (18%)	0.80
	Pseudomonas aeruginosa	7 (14%)	8 (12%)	0.99
	Sterile	0 (0%)	0 (0%)	N/A

**Table 3:** Assessment of parameters at day 5

Parameters		Group 1 (n=48) (mean ± SD)	Group 2 (n=49) (mean ± SD)	P-value
TLC (× 10^3^/mm^3^)		15.43 ± 1.67	17.27 ± 2.27	0.001*
Serum urea (mg/dL)		42.67 ± 11.05	46.84 ± 12.61	0.17
Serum creatinine (mg/dL)		0.76 ± 0.17	0.87 ± 0.27	0.25
Serum Na^+ ^(mmol/L)		139.42 ± 4.71	138.43 ± 4.14	0.28
Serum K^+ ^(mmol/L)		4.35 ± 0.43	4.22 ± 0.46	0.34
24-hour urine output (mL)		1745.21 ± 267.33	1723.27 ± 323.98	0.72
Endotracheal culture	Acinetobacter baumannii	30 (62.5%)	33 (67.3%)	0.67
	Klebsiella pneumoniae	7 (14.5%)	8 (16.3%)	0.99
	Pseudomonas aeruginosa	6 (12.5%)	8 (16.3%)	0.77
	Sterile	5 (10.4%)	0	0.03*

**Table 4:** Assessment of parameters at day 10

Parameters		Group 1 (n=48) (mean ± SD)	Group 2 (n=46) (mean ± SD)	P-value
TLC (× 10^3^/mm^3^)		11.84 ± 1.71	14.08 ± 1.94	<0.001
Serum urea (mg/dL)		44.23 ± 11.23	51.83 ± 13.69	0.04
Serum creatinine (mg/dL)		0.81 ± 0.27	1.002 ± 0.35	0.03
Serum Na^+ ^(mmol/L)		138.40 ± 4.65	136.74 ± 3.79	0.06
Serum K^+ ^(mmol/L)		4.41 ± 0.44	4.24 ± 0.51	0.27
24-hour urine output (mL)		1696.67 ± 256.35	1590.65 ± 302.14	0.06
Endotracheal culture	Acinetobacter baumannii	8 (17.8%)	20 (45.5%)	0.012
	Klebsiella pneumoniae	3 (6.7%)	7 (15.9%)	0.197
	Pseudomonas aeruginosa	2 (4.4%)	5 (11.4%)	0.266
	Sterile	32 (71.1%)	12 (27.3%)	<0.001

**Table 5:** Assessment of parameters at day 14

Parameters		Group 1 (n=45) (mean ± SD)	Group 2 (n=44) (mean ± SD)	P-value
TLC (× 10^3^/mm^3^)		10.08 ± 1.36	11.42 ± 1.91	0.33
Serum urea (mg/dL)		47.42 ± 12.52	55.09 ± 14.00	0.02
Serum creatinine (mg/dL)		0.90 ± 0.30	1.15 ± 0.37	0.02
Serum Na^+ ^(mmol/L)		136.80 ± 4.10	134.41 ± 3.47	0.06
Serum K^+ ^(mmol/L)		4.52 ± 0.49	4.44 ± 0.57	0.46
24 hour urine output (mL)		1567.11 ± 269.69	1381.36 ± 353.21	0.01
Endotracheal culture	Acinetobacter baumannii	3 (6.7%)	5 (11.4%)	0.49
	Klebsiella pneumoniae	1 (2.2%)	2 (4.5%)	0.62
	Pseudomonas aeruginosa	1 (2.2%)	1 (2.3%)	1.0
	Sterile	40 (88.9%)	36 (81.8%)	0.35

## Discussion

In this prospective study analysing 100 patients, it was found that for both groups, A. baumanii was the most common organism reported, followed by *K. pneumoniae* and *P. aeruginosa*. We observed a faster onset of microbiological eradication when aerosolized colistin is given adjunctively to the intravenous route as compared to intravenous alone; however, cure rates were comparable and statistically not significant. This could be due to the fact that the MIC required for microbiological eradication at the site of infection was achieved faster when nebulized colistin was given along with systemic therapy. Due to the poor lung penetration of intravenous colistin and colistin being a concentration-dependent antibiotic, it was easier to infer why bacteriological strains were massively eradicated faster with inhaled colistin adjunctive therapy. According to numerous studies, colistin inhaled is typically well tolerated, with only a small number of bronchoconstriction instances and no rise in the frequency of major systemic side effects such as nephrotoxicity [9]. *A. baumannii*-colonised patients with pneumonia had 75% microbiological success rate, according to Hsieh et al. [10]. The bacterial eradication rates with intravenous colistin treatment and inhaled plus systemic colistin treatment were 69% and 76%, respectively, in patients with VAP caused by *A. baumannii* [11]. Similar to this, Kuo et al. discovered that breathing colistin had a favourable impact on microbiological success and named it an independent predictor of eradication [12]. Abedellatif et al. observed that aerosolized colistin was as effective as intravenous colistin in the therapy of MDR bacilli VAP. Moreover, on analysis, improvements in oxygenation (i.e., P/F ratio) and a faster bacterial eradication time were also noted [13]. Boisson et al. found that after nebulization of 2 MIU of colistin in critically ill patients, colistin concentrations in the epithelial lining fluid of the lungs were very high (>100 mg/L in most cases), whereas plasma concentrations were low (<1 mg/L) [2].

We noted a significant reduction in TLC on the 5th and 10th days in group 1 as compared to group 2. Carillo et al. reported improvement in CRP levels and TLC after administration of inhaled colistin in MDR-gram-negative infection in post-lung transplant patients [14]. Similar observations were also reported by Moghaddam et al., which indicated a significant fall in TLC following the administration of inhaled colistin [15]. A decrease in TLC indicates resolution of infection, which was further consistent with endotracheal aspirate cultures.

It was observed that from the 10th day on, a significant increase in serum urea as well as serum creatinine level was recorded in patients receiving only IV colistin alone as compared to patients receiving colistin both IV and by nebulization. However, none of the patients showed electrolyte abnormalities, landed in acute renal failure (ARF), or required renal replacement therapy (RRT), and antibiotic therapy was not discontinued till the end of the 14th day. No statistically significant findings regarding serum sodium or serum potassium levels were noted.

From the 8th day onwards till the end of therapy on the 14th day, a decrease in urine output was noticed in patients receiving IV colistin alone as compared to those receiving both nebulized and IV colistin, which was statistically significant, but the decrease was not significant enough to be categorised under oliguria (urine output <400 ml/day) or acute kidney injury.

The D-amino-amino and fatty acid components of colistin contribute to dose-dependent renal toxicity, which is less than other polymyxins, especially polymyxin B [16]. According to Dalfino et al., no correlation could be found between variations in serum creatinine levels and the dose or duration of colistin therapy. AKI developed in 18% of patients receiving high-dose colistin (9 MIU twice-daily) did not require RRT and resolved within 10 days of colistin discontinuation [17]. Dewan and Shoukat found AKI in 16% of patients in a different study who received high dosage, prolonged interval colistin (9 MIU stat followed by 4.5 MIU 12 hr); however, no patient needed RRT [18]. A study conducted by Tumbarello et al. that evaluated the efficacy and safety of aerosolized plus IV colistin versus IV colistin alone in patients with VAP suggested that aerosolized colistin might be a beneficial adjunct to IV colistin in the management of VAP caused by MDR gram-negative bacilli susceptible to colistin [19]. Demirdal et al., based on RIFLE criteria, reported that the incidence of AKI was higher in patients receiving only IV colistin as compared to patients receiving nebulized colistin along with IV colistin [20]. Our findings are supported by Abdellatif et al., who also reported that the development of ARF was significantly lower with the administration of aerosolized colistin as compared with patients in the IV group. Also, the requirement for replacement renal therapy (RRT) was reduced in the aerosolized group [13]. Nebulized colistin has significantly reduced nephrotoxicity, as reported by Kim et al., which was consistent with our study [21].

In this study, we found no significant difference in the mean length of ICU stay between the two groups. Vardakas et al. concluded that no significant difference was present regarding the length of the ICU stay between nebulized and IV colistin in comparison with IV colistin alone [22]. No significant reduction in the duration of ICU stay was reported by Moghaddam et al. in patients receiving nebulized as well as IV colistin compared to IV colistin alone [15]. No significant difference was noted in 28-day ICU mortality between inhaled plus systemic colistin and systemic colistin alone, which is supported by previous data [19,23,24].

The limitations of this study are that it has involved patients from only a single institution and has included only trauma-critical care patients. Moreover, the result could possibly vary with the use of different nebulizers. Hence, we sincerely feel that these results need further validation using a heterogeneous population, on a multicentric basis, and with the use of different nebulizers.

## Conclusions

For nosocomial pneumonia due to multi-drug-resistant gram-negative bacteria, inhaled colistin therapy in addition to intravenous colistin therapy represents a promising approach owing to its systemic side effects compared to intravenous colistin treatment alone. Our results suggest that the combination of inhaled colistin and intravenous colistin has therapeutic benefits in terms of faster bacteriological cure and reduced nephrotoxicity.
